# Brother of Regulator of Imprinted Sites (BORIS) suppresses apoptosis in colorectal cancer

**DOI:** 10.1038/srep40786

**Published:** 2017-01-18

**Authors:** Yanmei Zhang, Mengdie Fang, Yongfei Song, Juan Ren, Jianfei Fang, Xiaoju Wang

**Affiliations:** 1 Center for Molecular Medicine, Zhejiang Academy of Medical Science, Hangzhou, Zhejiang Province, 310012, P.R. China

## Abstract

Identifying oncogenes that promote cancer cell proliferation or survival is critical for treatment of colorectal cancer. The Brother of Regulator of Imprinted Sites (*BORIS*) is frequently expressed in most types of cancer, but rarely in normal tissues. Aberrantly expressed *BORIS* relates to colorectal cancer, but its function in colorectal cancer cells remains unclear. In addition, previous studies indicated the significance of cytoplasm-localized BORIS in cancer cells. However, none of them investigated its function. Herein, we investigated the functions of BORIS in cancer cell proliferation and apoptosis and the role of cytoplasm-localized BORIS in colorectal cancer. *BORIS* expression correlated with colorectal cancer proliferation. BORIS overexpression promoted colorectal cancer cell growth, whereas *BORIS* knockdown suppressed cell proliferation. Sensitivity of colorectal cancer cells to 5-fluorouracil (5-FU) was inversely correlated with *BORIS* expression. These data suggest that BORIS functions as an oncogene in colorectal cancer. *BORIS* silencing induced reactive oxygen species (ROS) production and apoptosis, whereas BORIS supplementation inhibited apoptosis induced by *BORIS* short interfering RNA (siRNA), hydrogen peroxide (H_2_O_2_) or 5-FU. Introduction of BORIS-ZFdel showed that cytoplasmic localization of BORIS inhibited apoptosis but not ROS production. Our study highlights the anti-apoptotic function of BORIS in colorectal cancer.

Colorectal cancer is the third most common type of cancer in the world^1^,^2^. Despite advances in colorectal cancer research and treatment, colorectal cancer remains incurable because of drug resistance^1^,^2^,^3^. Personalized treatment has the potential to increase the efficacy and decrease toxicity. However, prognostic and predictive markers that show promise in the clinic are required for targeted therapies. The Brother of Regulator of Imprinted Sites (*BORIS*) is the paralogue of CCCTC-binding factor (*CTCF*)^4^,^5^. In contrast to *CTCF*, which is expressed universally in all somatic and germ cells, *BORIS* is specifically expressed in the embryo, skin, germ cells, and cancer, including colorectal cancer^4^,^6^,^7^,^8^,^9^,^10^,^11^,^12^,^13^. In colorectal cancer, the copy number of *BORIS* is amplified and *BORIS* is aberrantly expressed^6^,^9^,^14^, suggesting the potential clinical significance of BORIS in the diagnosis/treatment of colorectal cancer.

BORIS is required for cell proliferation in certain types of cancer^15^,^16^,^17^,^18^,^19^. In breast cancer, silencing of *BORIS* by short interfering RNA (siRNA) suppressed cancer cell viability and induced caspase 3/7 activity^15^. These findings suggest that aberrant expression of *BORIS* might suppress apoptosis in cancer cells. Abnormal expression of *BORIS* was detected in colorectal cancer^6^,^9^. BORIS may destroy the balance between anti-apoptotic and proapoptotic effectors, followed by inhibition of apoptosis and decreased colorectal cancer cell death. Whether *BORIS* is required for proliferation and/or inhibits apoptosis of colorectal cancer cells remains undetermined.

BORIS is localized in both nucleus and cytoplasm to various extents in carcinoma^20^. In the nucleus, BORIS serves as a scaffold to regulate gene expression^21^,^22^. In the cytoplasm, BORIS binds to RNA and associates with actively translating ribosomes^23^. Both cytoplasm- and nucleus-localized BORIS are related to prostate cancer^24^. BORIS might thus have certain functions in the cytoplasm. However, no studies have tested this hypothesis.

In this study, we evaluated the association of BORIS and cytoplasm-localized BORIS with clinical colon cancer. We identified the requirement of *BORIS* in colorectal cancer and provided direct evidence that zinc finger domains (ZF domains) deleted and cytoplasm-localized BORIS-ZFdel suppressed apoptosis. While we observed that BORIS-ZFdel did not restore the cell death triggered by 5-fluorouracil (5-FU) or hydrogen peroxide (H_2_O_2_) and did not inhibit the reactive oxygen species (ROS) production induced by *BORIS* silencing. The discovery of the cytoplasmic roles of BORIS in apoptotic inhibition highlights the potential clinical applications of BORIS for the treatment of colorectal cancer.

## Results

### *BORIS* expression correlates with colorectal cancer

**Figure 1:**
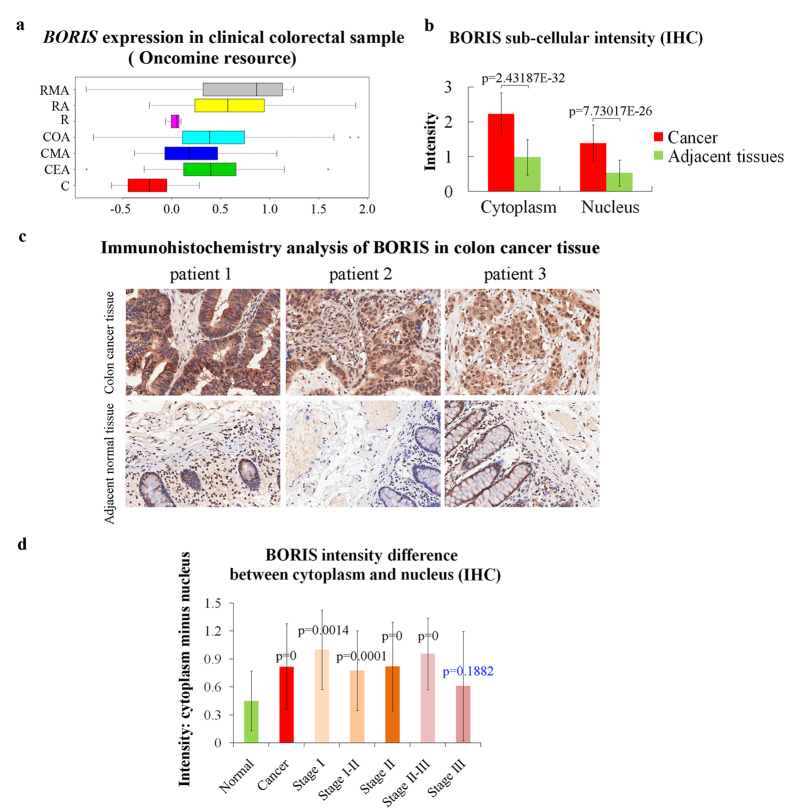
Abnormal expression of *BORIS* in clinical colorectal cancer tissues. (**a**) Data downloaded from Oncomine database were replotted by R.3.2.3 boxplot. The Y axis indicates colorectal tissues. RMA: Rectal mucinous adenocarcinoma (N = 6), RA: rectal adenocarcinoma (N = 60), R: rectum (N = 3), COA: colon adenocarcinoma (N = 102), CMA: colon mucinous adenocarcinoma (N = 20), CEA: cecum adenocarcinoma (N = 24), C: Colon (N = 19). The normal colon and rectal cases were used as controls (defined as h) to calculate the differential expression of *BORIS* between cancer and normal tissues. The p-values between cancer and normal samples were calculated using the Wilcoxon test and were 0.01186 (h, RMA), 1.017e-09 (h, RA), 1.89e-08 (h, COA), 0.000226 (h, CMA), and 2.897E-06 (h, CEA). (**b**) Statistical analysis of BORIS intensity difference between cancer and adjacent tissues detected by IHC in 180 FFPE clinical colon tissues. (**c**) Representative IHC images captured using a 10×objective. (**d**) Statistical analysis of the difference in the content of BORIS between the cytoplasm and nucleus in clinical colon tissues.

**Figure 2:**
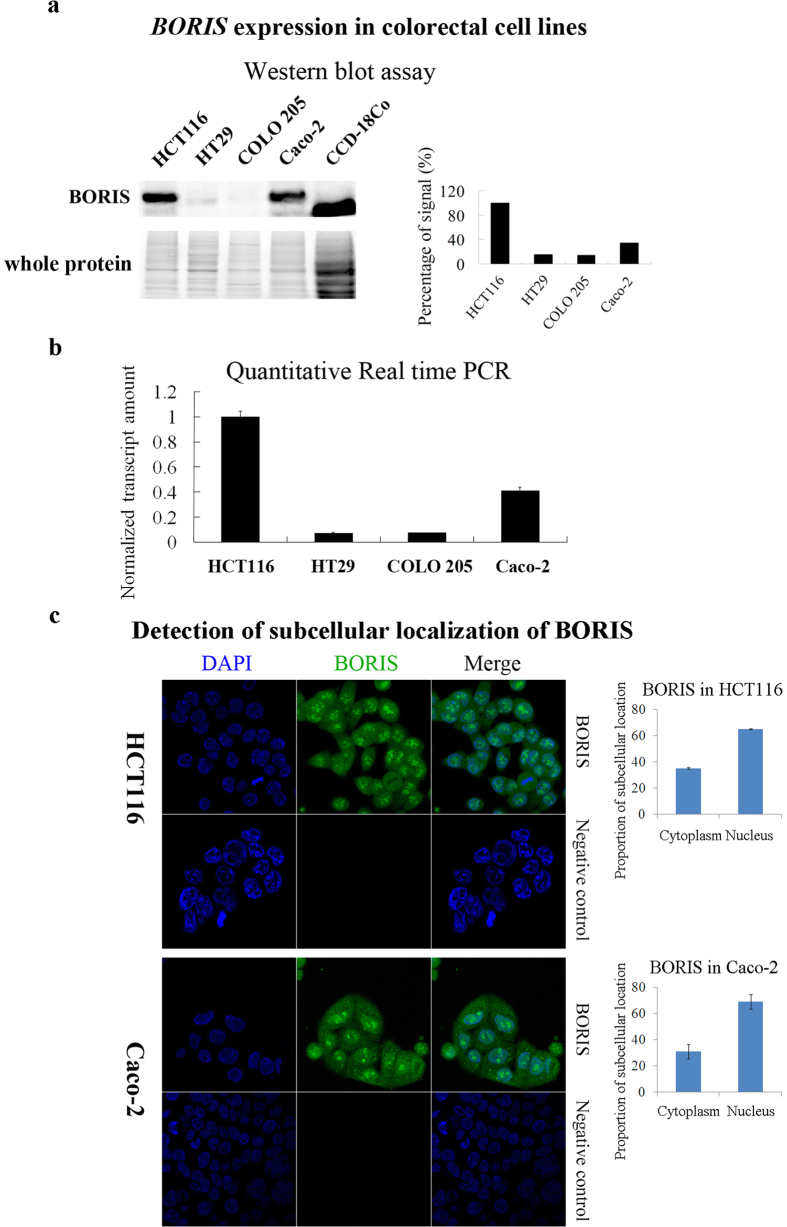
Expression and sub-cellular localization of BORIS in colorectal cancer cell lines. (**a**) *BORIS* expression levels in colorectal cell lines were determined by western blot assay in the left panel, which were shown on cropped blots. Full-length blots and whole protein on nitrocellulose membrane were included in Supplementary Fig. S1a. The graph in the right panel represents the percentage of the signal in each fraction measured by densitometric analysis of the western blot. (**b**) *BORIS* expression levels in colorectal cancer cell lines were determined by using quantitative real-time PCR. (**c**) Localization of endogenous BORIS in HCT116 and Caco-2 cells. The right panels show the proportion of the signal distributed in the cytoplasm and in the nucleus.

Analysis of data extracted from the Oncomine database revealed a high correlation between *BORIS* expression and colorectal cancer. *BORIS* expression in different types of colorectal cancer varied, but was significantly different in all colorectal cancer types compared to that in normal tissues (Fig. 1a). To further verify the significance of BORIS in colorectal cancer, the expression and sub-cellular localization of BORIS were observed by immunohistochemistry (IHC) assay in 180 clinical colon samples, which included 100 cancer tissues and 80 adjacent normal tissues collected from 100 colon cancer patients (Table 1). BORIS was expressed at higher levels in cancer than in adjacent normal tissues in both the nucleus and cytoplasm (Fig. 1b and c). BORIS was localized more in the cytoplasm than in the nucleus in colon cells (Fig. 1b). The difference in BORIS contents between the cytoplasm and nucleus was calculated. The content of cytoplasmic BORIS was significantly increased in colon cancer compared to adjacent normal cells (Fig. 1d). To confirm these observations, *BORIS* expression and sub-cellular localization were assessed in four colorectal cancer cell lines: a colorectal adenocarcinoma cell line (Caco-2) and 3 colorectal carcinoma cell lines (COLO 205, HT29, and HCT116), together with one normal colon-derived fibroblast cell line (CCD-18Co). We did not detect BORIS in CCD-18Co cells, but only a truncated protein (Fig. 2a). However, we detected different levels of *BORIS* expression in all four colorectal carcinoma cell lines. The highest expression was detected in HCT116 cells, followed by Caco-2, HT29, and COLO 205cells (Fig. 2a,b and Supplementary Fig. S1a). In colorectal cancer cell lines, BORIS was detected more in the nucleus than in the cytoplasm (Fig. 2c and Supplementary Fig. S1b). This is different from the observation in clinical samples that BORIS was localized more in the cytoplasm than in the nucleus (Fig. 1b). This discrepancy might have resulted from the different methods of detection between formalin-fixed paraffin-embedded (FFPE) samples and freshly fixed cells.

### *BORIS* is required for colorectal cancer cell proliferation

**Figure 3:**
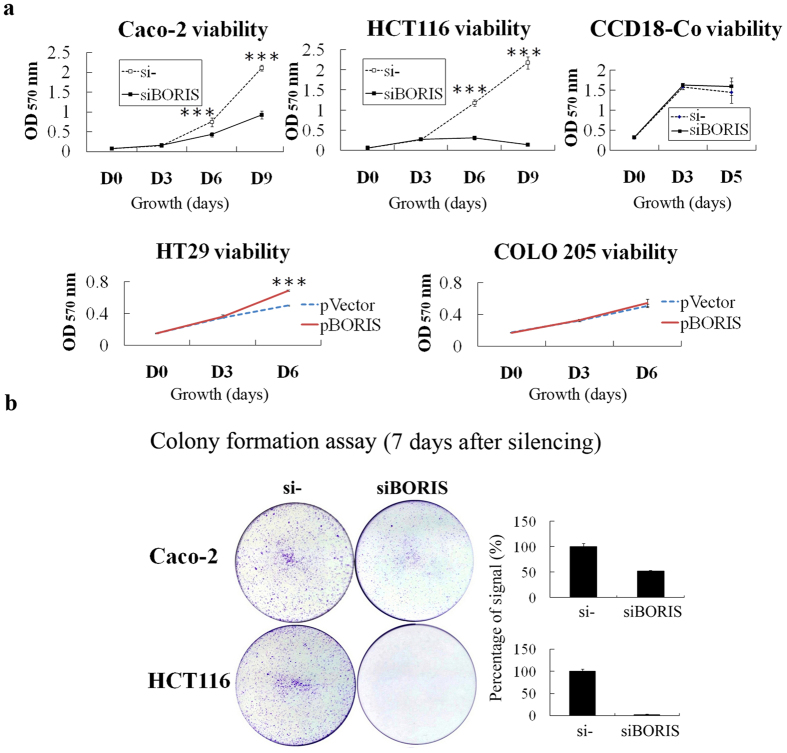
*BORIS* expression affected colorectal cancer cell proliferation. (**a**) Colorectal cell viability was determined by using the MTT assay. Dashed lines indicate the growth of control siRNA-and pVector-transfected cells. Solid lines indicate the growth of *BORIS* siRNA-and pBORIS-transfected cells. (**b**) The colony formation ability of two colorectal cancer cell lines was assessed by crystal violet staining 7 days after siRNA transfection. The right panel indicates the percentage of signal in each well compared to that in the control siRNA-transfected cells. Statistical differences between the control and treatments were evaluated by two-tailed Student’s t-test. ***p < 0.001.

To study the function of *BORIS* in the proliferation of colorectal cancer, *BORIS* was silenced in Caco-2 and HCT116 cells by siRNA and overexpressed in HT29 and COLO 205 cells. The sequence targeted by *BORIS* siRNA (siBORIS) is shown in Supplementary Fig. S2a 15. The silencing efficiency was assessed by western blot and quantitative real time PCR (Supplementary Fig. S2b and S2c). In both Caco-2 and HCT116 cells, the cell viability was remarkably decreased by *BORIS* silencing (Fig. 3a). *BORIS* silencing dramatically inhibited the colony formation ability of Caco-2 cells (Fig. 3b top panel). No colonies were observed after *BORIS* silencing in HCT116 cells (Fig. 3b bottom panel). These results clearly show that *BORIS* is required for the survival of colorectal cancer cells. HT29 and COLO 205 are two colorectal cancer cell lines with low levels of BORIS. BORIS overexpression promoted the proliferation of HT29 cells but not COLO 205 cells at six days.

### *BORIS* silencing induces apoptosis in colorectal cancer cells

**Figure 4:**
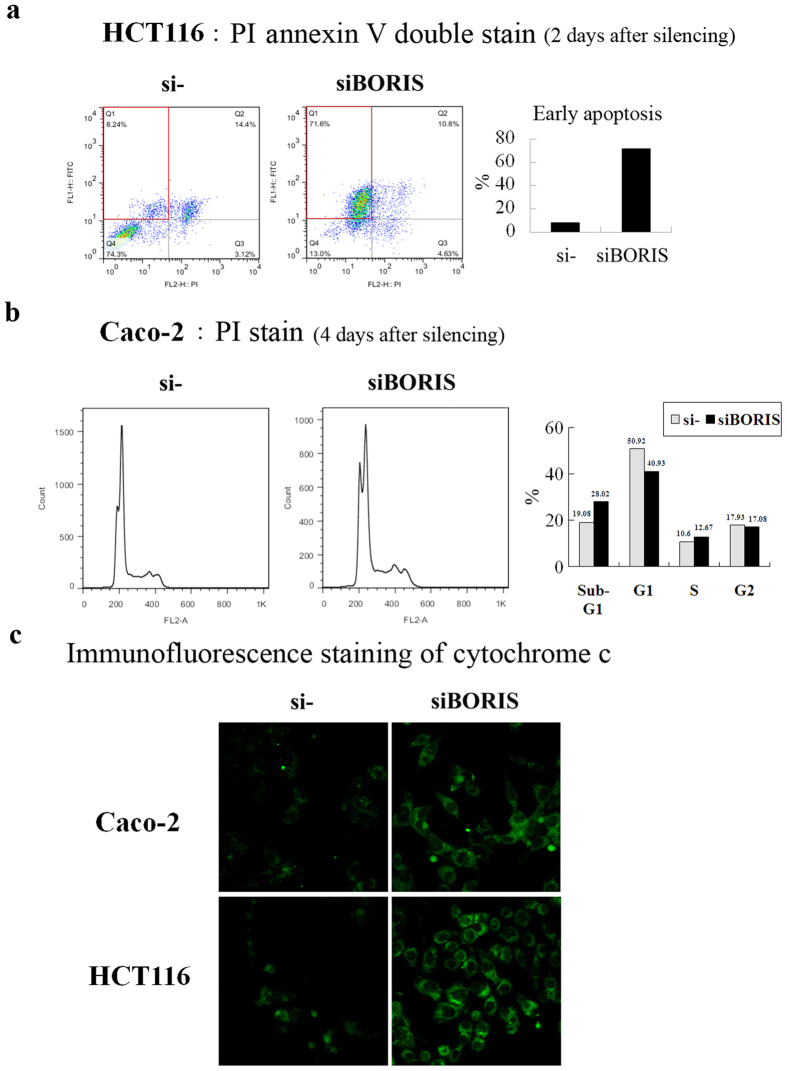
*BORIS* silencing induced apoptosis of colorectal cancer cells. (**a**) PI and annexin V double staining indicates apoptotic cells. The right panel indicates the percentage of early apoptotic cells under each treatment. (**b**) PI single staining of *BORIS*-silenced Caco-2 cells indicated an increase of the sub-G1 peak. (**c**) Cytochrome c immunofluorescence staining of *BORIS*-silenced Caco-2 and HCT116 cells. The secondary antibody conjugated with FITC indicated the location of cytochrome c.

*BORIS* silencing caused HCT116 cell death and Caco-2 cell growth suppression (Fig. 3). Given that *BORIS* siRNA induces apoptosis in breast cancer^15^, we investigated whether *BORIS* silencing suppresses colorectal cancer cell growth by inducing apoptosis. We used flow cytometry and cytochrome c release assays to test this hypothesis. Apoptosis was detected by annexin V and propidium iodide (PI) double staining in HCT116 cells 2 days post *BORIS* silencing (Fig. 4a). PI single staining showed an increase in the sub-G1 peak in DNA content histograms for Caco-2 cells at 4 days post *BORIS* silencing (Fig. 4b). The cells did not show apparent cell cycle arrest (Fig. 4b). Cytoplasmic cytochrome c was detected in *BORIS*-silenced HCT116 and Caco-2 cells (Fig. 4c and Supplementary Fig. S3). All of these results consistently indicate that *BORIS* silencing inhibits colorectal cancer cell proliferation by inducing apoptosis.

### Cytoplasm-localized BORIS suppresses apoptosis

**Figure 5:**
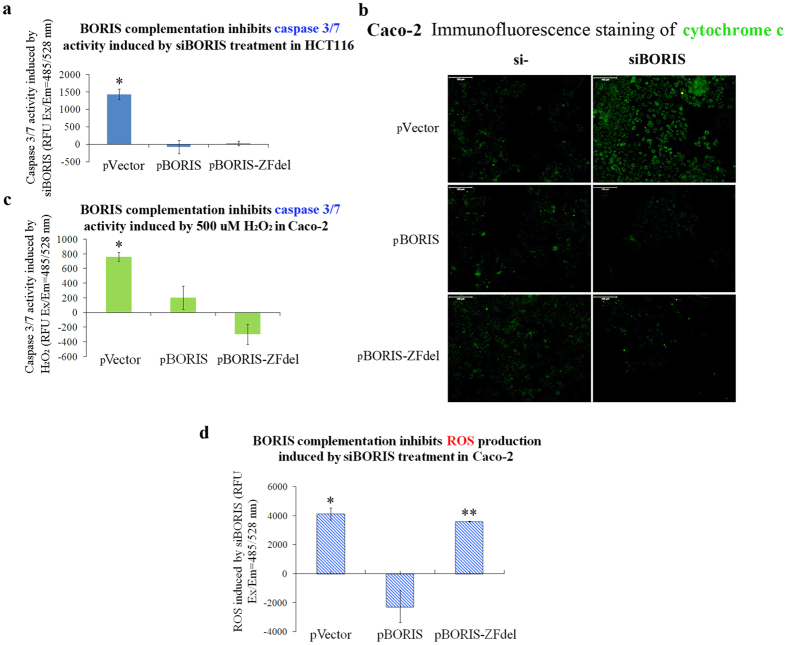
BORIS and cytoplasm-localized BORIS-ZFdel suppressed apoptosis and *BORIS* silencing induced ROS formation. (**a**) Caspase 3/7 activity induced by *BORIS* silencing was reduced by transfection with plasmids expressing BORIS or BORIS-ZFdel. The empty vector was used as an overexpression control. The Y axis indicates the difference in caspase 3/7 activity between *BORIS* siRNA- and negative control siRNA**-**transfected cells. (**b**) Transfection of pBORIS and pBORIS-ZFdel suppressed the release of cytochrome c induced by *BORIS* silencing. Cytochrome c was immunostained by using a mouse monoclonal cytochrome c antibody and FITC-conjugated secondary antibody. (**c**) Caspase 3/7 activity induced by H_2_O_2_ treatment was suppressed by either BORIS or BORIS-ZFdel overexpression. The Y axis indicates the difference in caspase 3/7 activity between H_2_O_2_ and H_2_O treated cells. (**d**) ROS production induced by siBORIS was suppressed by overexpression of BORIS, but not BORIS-ZFdel. The Y axis indicates induction of ROS by siBORIS treatment. Statistical differences between the control and treatments were evaluated by two-tailed Student’s t-test. *p < 0.05; **p < 0.01.

BORIS is localized in both cytoplasm and nucleus (Figs 1c and 2c and Supplementary Fig. S1b). The content of cytoplasmic BORIS increases in colon cancer tissue (Fig. 1d). To explore the function of the cytoplasm-localized BORIS, we constructed a cytoplasm-localized BORIS by deleting the zinc finger domains, which contain two nuclear localization sequences (NLS) (Supplementary Figs S4 and S5). The cytoplasmic form of BORIS-ZFdel was transfected into cells treated with *BORIS* siRNA (Supplementary Fig. S2a). The activity of caspase 3/7 was attenuated and the leakage of cytochrome c from the mitochondria to the cytosol was blocked by the expression of BORIS-ZFdel (Fig. 5a and b). These results indicate that complementation with BORIS-ZFdel inhibited apoptosis.

To test whether cytoplasm-localized BORIS suppressed apoptosis selectively, we examined the effect of BORIS-ZFdel on apoptosis triggered by an apoptosis inducer, H_2_O_2_, which increases mitochondrial permeability and release of cytochrome c^25^. Overexpression of either the full-length BORIS or truncated BORIS-ZFdel suppressed caspase 3/7 activity induced by H_2_O_2_ treatment (Fig. 5c), indicating that cytoplasm-localized BORIS did not inhibit apoptosis selectively. Therefore, ectopic overexpression of BORIS or BORIS-ZFdel might inhibit the apoptotic cascade by suppressing ROS production in colorectal cancer cells. However, ROS production induced by *BORIS* silencing was only inhibited by BORIS and not by BORIS-ZFdel (Fig. 5d). Cytoplasm-localized BORIS might inhibit apoptosis of colorectal cancer cells through unknown mechanism unrelated to resistance to ROS.

### The sensitivity of colorectal cancer cells to 5-FU is affected by BORIS expression

**Figure 6:**
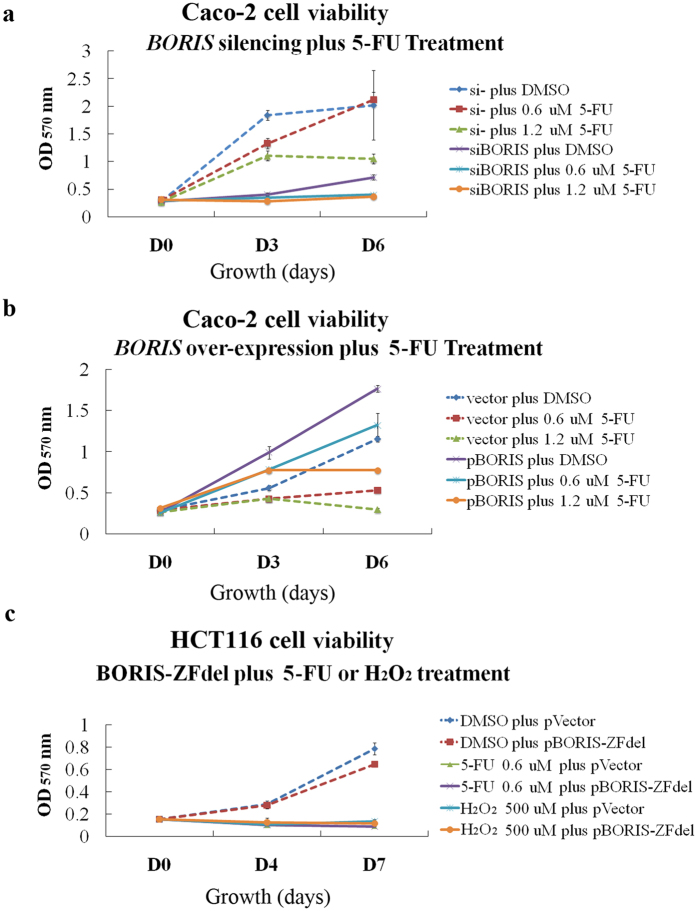
Sensitivity of colorectal cancer cells to 5-FU is affected by BORIS expression. (**a**) Caco-2 cells were treated with BORIS siRNA together with 5-FU. The viability of the treated cells was evaluated. Negative control siRNA and DMSO-treated cells were used as controls. (**b**) The cell viability of *BORIS*-overexpressing Caco-2 cells treated with 5-FU was compared with that of controls. (**c**) Overexpression of pBORIS-ZFdel did not counteract the inhibitory effect of 5-FU or H_2_O_2_ on the proliferation of HCT116 cells. The statistical differences between the samples are evaluated by two-tailed Student’s t-test and presented in Supplementary Fig. S9. *p < 0.05; **p < 0.01; ***p < 0.001. The transfection efficiency of the siRNA and the plasmids is shown in Supplementary Fig. S10.

The chemotherapy drug 5-FU is the first choice for the treatment of colorectal cancer^26^,^27^. We evaluated the effect of 5-FU treatment on colorectal cancer cells with low or high *BORIS* expression levels. Given that *BORIS* knockdown is sufficient to kill HCT116 cells, Caco-2 cells, which express BORIS at a moderate level, were studied under 5-FU treatment. *BORIS* knockdown in Caco-2 cells resulted in a growth inhibition even stronger than that observed on treatment with 1.2 μM 5-FU alone (IC50 for Caco-2 cells), indicating that *BORIS* silencing strongly inhibited cell growth (Fig. 6a). When siBORIS-transfected Caco-2 cells were treated with 1.2 μM 5-FU, the cell growth was arrested, suggesting a synergistic effect of the combination treatment (Fig. 6a). Interestingly, a similar synergistic effect was observed in si*BORIS-*transfected Caco-2 cells treated with a lower dose of 5-FU (0.6 μM) (Fig. 6a). These results suggest that *BORIS* silencing could enable the 5-FU dose to be reduced in the treatment of colorectal cancer.

We further examined the correlation between *BORIS* expression and the proliferation of colorectal cancer cells. In particular, we overexpressed *BORIS* in Caco-2 cells and examined its effect on the proliferation of colorectal cancer cells. *BORIS* overexpression increased the cell viability of Caco-2 cells and attenuated the sensitivity of cancer cells to 5-FU treatment (Fig. 6b).

**Figure 7:**
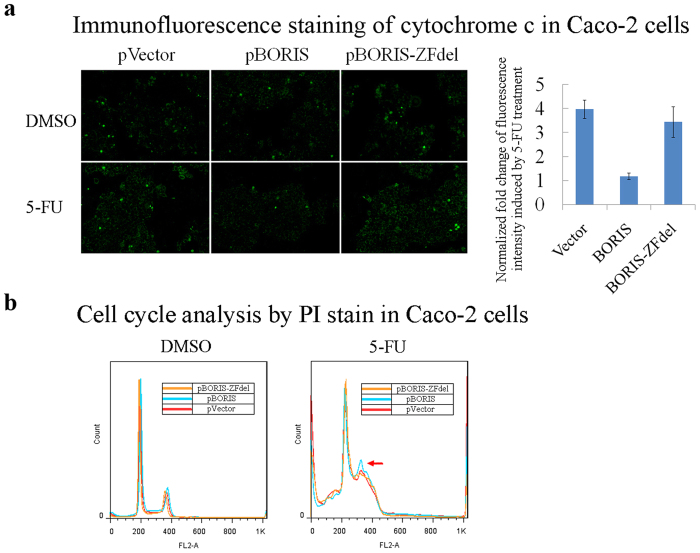
BORIS but not BORIS-ZFdel inhibits apoptosis induced by 5-FU. (**a**) Cytochrome c immunofluorescence staining of 5-FU-treated Caco-2 cells with overexpression of pVector, pBORIS or pBORIS-ZFdel. The secondary antibody conjugated with FITC indicated the release of cytochrome c. The right panel indicates the fold change of immunofluorescence staining signals. (**b**) PI staining of the DNA content indicates difference in cell cycles under treatment with 5-FU.

Interestingly, BORIS overexpression, but not that of BORIS-ZFdel, attenuated the effect of 5-FU or H_2_O_2_ on the proliferation of colorectal cancer cells (Fig. 6b and c). BORIS but not BORIS-ZFdel inhibited cytochrome c release induced by 5-FU treatment (Fig. 7a). Cell cycle analysis by PI staining of DNA content showed that BORIS overexpression protectd DNA synthesis under treatment with 5-FU (Fig. 7b). These data suggest that the deleted zinc finger domains of BORIS might play an important role in resistance against the effects of 5-FU or H_2_O_2_.

### BORIS supplementation does not restore the suppression of *c-Myc* caused by *BORIS* silencing

**Figure 8:**
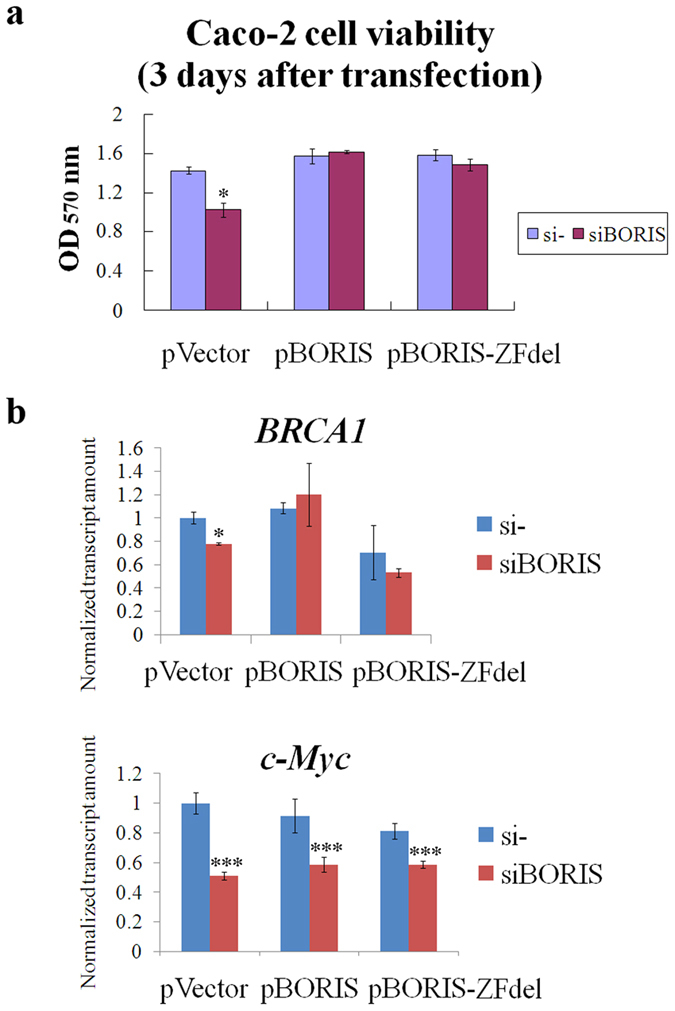
BORIS supplementation restores cell proliferation inhibition but not the suppression of *c-Myc* caused by *BORIS* silencing. (**a**) BORIS or BORIS-ZFdel complementation restored the cell viability suppressed by siBORIS. (**b**) BORIS and BORIS-ZFdel restored the expression of *BRCA1* but not *c-Myc*, which were suppressed by siBORIS. Statistical differences between the si- control and siBORIS treatments were evaluated by two-tailed Student’s t-test. *p < 0.05, ***p < 0.001.

BORIS is reported to affect the expression of downstream genes such as *BRCA1* and *c-Myc* by demethylating their promoters^21^,^22^,^28^. Thus, demethylation may be the mechanism by which BORIS promotes the oncogenesis. In the present study, 5-Aza-2′-deoxycytidine (5-Aza-dc) treatment up-regulated *BORIS*, but the elevated BORIS level did not reverse the damage caused by demethylation of the entire genome (Supplementary Fig. S6). The expression of *BRCA1* and *c-Myc* was then examined by altering the expression of *BORIS*. BORIS and cytoplasmically localized BORIS-ZFdel reversed the cell proliferation inhibition and *BRCA1* suppression caused by *BORIS* silencing (Fig. 8), whereas they did not induce the expression of *c-Myc* (Fig. 8b). Considering the function of cytoplasm-localized BORIS-ZFdel in resisting apoptosis, it could not promote cell proliferation by epigenetic regulation directly. We speculate that BORIS-ZFdel affects cell proliferation and apoptosis by cytoplasmic signaling pathways.

## Discussion

In our study, BORIS was not detected in the normal fibroblast colon cell line, CCD-18Co. However, a truncated protein was detected using the monoclonal antibody that targets the N-terminal of BORIS (Fig. 2a). We used *BORIS* siRNA to examine the function of this protein in CCD-18Co cells. *BORIS* siRNA did not decrease CCD-18Co cell viability (Fig. 3a). The sequence targeted by *BORIS* siRNA in CCD-18Co cells contains no mutations (Supplementary Fig. S7). We concluded that the truncated protein in CCD-18Co cells did not have function demonstrated for BORIS in Caco-2 and HCT116 cells. Comparing the sequence of the *BORIS* detected in CCD-18Co with that in Caco-2 or HCT116 cells in future investigations would reveal key functional elements of *BORIS* for promotion of colorectal cancer proliferation.

We determined that subtypes of clinical colorectal cancer samples and colorectal cancer cell lines showed differential *BORIS* expression and different responses to *BORIS* alternation (Figs 1a, 2a,b, 3a and b). Alberti *et al*. also observed different behaviors of tumor cells upon aberrant expression of BORIS^29^. The isoform variants of BORIS may function differently in different cell backgrounds^30^,^31^. Colorectal cancer is a heterogeneous multi-stage disease. *BORIS* expression may indicate overlapped or mixed expression of *BORIS* variants in a few stages of cancer and may be used as marker for colorectal cancer. *BORIS* silencing in colorectal cancer cells highly expressing BORIS strongly suppressed cancer cell growth compared with that in colorectal cancer cells with low BORIS expression, suggesting the potential of *BORIS* knockdown for treatment of colorectal cancer with high expression of BORIS.

We noticed that *BORIS* silencing increased ROS production in colorectal cancer cells, whereas ectopic overexpression of BORIS but not cytoplasmic BORIS-ZFdel suppressed the ROS production (Fig. 5d). It suggests that the ROS production caused by the lack of nuclear BORIS is not restored by the cytoplasmic BORIS-ZFdel. This hypothesis is supported by the finding that *BORIS* silencing increased the inhibition of colorectal cancer cell growth by 5-FU (Fig. 6a), which inhibits DNA synthesis and increases the production of ROS^32^, and that overexpression of *BORIS* but not *BORIS-ZFdel* attenuated the suppressive effect of 5-FU on colorectal cancer cell growth and cytochrome c release (Figs 6 and 7). The expression level and sub-cellular localization of *BORIS* may be applied for predicting the outcome of colorectal cancer therapy.

We speculate that BORIS-ZFdel affects cell proliferation and apoptosis by cytoplasmic signaling pathways. Two apoptosis pathways have been documented: the extrinsic death receptor signaling pathway, which is triggered from the cell membrane, and the intrinsic mitochondria-mediated pathway, which is regulated by members of the Bcl-2 family^33^,^34^. We observed that the release of cytochrome c from mitochondria was blocked by BORIS-ZFdel (Fig. 5b and Supplementary Fig. S3), suggesting that the intrinsic mitochondria-mediated apoptosis pathway may be involved in the inhibition of apoptosis in colorectal cancer cells. BORIS may recruit proteins (e.g., Bcl-2 and VDAC) involved in the formation of mitochondrial permeability transition pores, considering that BORIS has been reported to act as a scaffold upon which BAT3 and SET1A assemble and bind to the upstream promoter regions of *c-Myc* and *BRCA1* in the nucleus^21^,^22^. Similarly, cytoplasm-localized BORIS may provide a platform for the assembly of apoptosis-related partners to inhibit apoptosis. The interaction between cytoplasm-localized BORIS and candidate partners and the co-localization between BORIS and mitochondria need to be further investigated.

In conclusion, the data presented here indicate that aberrant expression of *BORIS* inhibits apoptosis, promotes proliferation, and attenuates the sensitivity of colorectal cancer cells to 5-FU treatment. Mechanistic studies demonstrated that *BORIS* silencing induces ROS and apoptosis. Following complementation with the cytoplasm-localized BORIS-ZFdel, apoptosis induced by H_2_O_2_ or *BORIS* silencing was inhibited in colorectal cancer cells. However, ROS production induced by *BORIS* silencing was not inhibited by BORIS-ZFdel, suggesting that cytoplasm-localized BORIS might inhibit apoptosis through unknown mechanism unrelated to resistance to ROS. Future studies should be designed to test the association between BORIS and apoptosis pathways. Taken together, our data indicate that BORIS has considerable clinical significance. Modulation of the expression and sub-cellular localization of BORIS in colorectal cancer cells may provide novel therapeutic strategies for colorectal cancer.

## Material and Methods

### Clinical colon tissues and IHC assay

In total, 100 colon cancer tissues and 80 adjacent normal tissues were collected from 100 colon cancer patients in Taizhou Hospital of Zhejiang province, P. R. China (Table 1). We confirmed that informed consent was obtained from all subjects. All experimental protocols were approved by licensing committee of Taizhou Hospital of Zhejiang province, P. R. China. Immunohistochemistry staining of BORIS was performed on a tissue array (SHANGHAI OUTDO BIOTECH CO., LTD, China). The intensity of the signal in the cytoplasm and nucleus was recorded. All methods were performed in accordance with guidelines and regulations of Zhejiang Academy of Medical Science.

### Cell culture

The colorectal cell lines used in this study included HCT116, Caco-2, Colo 205, HT29, and CCD-18Co cells. Cells were cultured in Dulbecco’s Modified Eagle Medium (DMEM) supplemented with 10% FBS.

### Transfection and drug treatment

Lipofectamine^®^ RNAiMAX was used for silencing according to manufacturer’s protocol. Lipofectamine^®^ 3000 (Thermo Fisher Scientific, Waltham, MA, USA) was used for ectopic overexpression. The pBORIS plasmid was purchased from OriGene Technologies (Rockville, MD, USA). pBORIS-ZFdel was constructed from pBORIS (Supplementary Fig. S4). Cells were plated on 6-well or 96-well plates one day before transfection or drug treatment. 5-FU was dissolved in DMSO and supplied in DMEM medium for treatment. The procedure for the experiments related to 5-FU treatment is presented in Supplementary Fig. S8. H_2_O_2_ (500 μM) was applied for 20 hours to induce apoptosis. 5-Aza-dc (5 μM) was applied twice to induce demethylation, with 50% acetic acid used as a negative control. The volume of all of the added reagents did not exceed 0.1%.

### Quantitative real-time PCR

RNA from the cell pellet was extracted using TRIzol^®^ (Thermo Fisher Scientific) and ethanol precipitation. After quantification using a Nanodrop 2000 system, equal amounts of RNA from control and treated samples were reverse transcribed to cDNA. The expression of candidate genes was quantified by real-time PCR using *GAPDH* and actin as internal control genes. The primers and siRNA used in this study are listed in Supplementary Table 1.

### Cell viability analysis

In total, 800 to 1,000 cells per well in 100 μL of culture medium were plated in the wells of 96 well-plates one day before further treatment. Five replicates were performed for each treatment. Thiazolyl blue tetrazolium bromide (MTT, 500 μg/mL) was added to assess the cell viability at each time point.

### Colony formation assay

Cells were fixed with 4% formaldehyde for 20 minutes at room temperature (between 25 °C and 30 °C) and rinsed twice with PBS. The cells were then stained with 0.1% crystal violet for 15 minutes. After removal of the crystal violet, the cells were washed gently with water. Images of the stained dry cells were captured.

### Flow cytometry (FACS) assay

Cells were collected two or four days after treatment and stained with annexin V-FITC and/or propidium iodide (PI). FACS was performed using BD FACS Calibur to detect apoptosis.

### Caspase 3/7 assay

Cells (800 to 1,000 cells per well) were plated in 96-well white plates. SiRNA was transfected one day after plating. Complementation of full-length or truncated *BORIS* was performed one day after silencing. Caspase 3/7 activity was examined 3 days after the complementation. Cells plated on 6-well and 24-well plates used for other assays received the same treatments.

### Immunofluorescence

Cells cultured on glass were fixed in 4% formaldehyde and permeabilized by 0.3% Triton X-100 in PBS for 10 min. The fixed cells were then blocked for 30 min in PBS containing 1% bovine serum albumin (PBS-BSA). Antibodies diluted in PBS-BSA were applied for an overnight incubation in 4 °C. After three washes with PBS, the secondary FITC-conjugated antibodies were applied for 1 hour at room temperature. DAPI was used to stain the nucleus. Images were captured by using a laser scanning confocal microscope or conventional microscope.

### ROS assay

Cells (800 to 1,000 cells per well) were plated in 96-well black plates. The pVector, pBORIS, and pBORIS-ZFdel plasmids were transfected using Lipofectamine^®^ 3000 one day after cell plating. siBORIS transfection was performed one day after overexpression. ROS production was examined 2 days after silencing according to the manufacturer’s protocol. ROS were detected using 2,7-dichlorodihydrofluorescein diacetate (DCFH-DA), which was purchased from Beyotime Biotechnology (S0033, Shanghai, P. R. China).

### Antibodies

The BORIS antibody was supplied by Santa Cruz Biotechnology (sc-377085, Santa Cruz, CA, USA). The Flag antibody was supplied by Sigma (F3165, St Louis, MO, USA). The cytochrome c antibody was purchased from Beyotime Biotechnology (Shanghai, P. R. China).

### Statistical analysis

All data were obtained in a minimum of triplicates and are expressed as the mean ± standard deviation (SD). Statistical differences between the control and treatments were evaluated by two-tailed Student’s t-test. P < 0.05 was considered statistically significant. The clinical expression data for *BORIS* were downloaded from Oncomine and replotted by R.3.2.3 boxplot. Normal colon and rectal cases were collected as controls (defined as h) to determine the differential expression between cancer and normal tissues. The p-value was calculated using the Wilcoxon test.

## Additional Information

**How to cite this article**: Zhang, Y. *et al*. Brother of Regulator of Imprinted Sites (BORIS) suppresses apoptosis in colorectal cancer. *Sci. Rep.* **7**, 40786; doi: 10.1038/srep40786 (2017).

**Publisher's note:** Springer Nature remains neutral with regard to jurisdictional claims in published maps and institutional affiliations.

## Supplementary Material

Supplementary Information

## Figures and Tables

**Table 1:** Characteristics of 100 patients suffering from colon cancer.

Characteristics	Patients (N = 100)	Stage				
		I (N = 4)	I-II (N = 24)	II (N = 46)	II-III (N = 16)	III (N = 10)
**Sex**						
Male	54	2	15	24	9	4
Female	45	2	9	22	6	6
not available	1				1	
**Age at diagnosis** (**year**)						
<60	25	2	6	13	4	2
> = 60	74	2	18	33	11	8
not available	1				1	
**Overall survival** (**month**)						
median	35.11 (3–97)	42.5 (30–93)	39.33 (11–97)	34.86 (3–97)	24.25 (1–96)	31 (5–95)
