# Supplementary material for: Brother of Regulator of Imprinted Sites (BORIS) suppresses apoptosis in colorectal cancer
Source: Sci Rep. 2017 Jan 18;7:40786. doi: 10.1038/srep40786 (PMC5241680; doi:10.1038/srep40786)
Supplement: Supplementary Information [file srep40786-s1.pdf]

Brother of Regulator of Imprinted Sites (BORIS) suppresses apoptosis in colorectal cancer

Yanmei Zhang*, Mengdie Fang, Yongfei Song, Juan Ren, Jianfei Fang and Xiaoju Wang*

Center for Molecular Medicine,Zhejiang Academy of Medical Science, Hangzhou, Zhejiang Province, 310012, P.R. China

*Corresponding authors. Tel.: +86 571 88215563;
E-mail addresses: yanmeizhang81@yahoo.com (Yanmei Zhang) and wang.x.george@gmail.com(Xiaoju Wang)

Supplementary Information

Supplemental Figure Legends

Supplementary Figure S1. (a) *BORIS* expression levels in colorectal cell lines. Full-length blot and whole protein on nitrocellulose membrane of Figure 2a. (**b**) Localization of endogenous BORIS in HT29 and COLO 205 cells. The right panels show the proportion of the signal distributed in the cytoplasm and in the nucleus.

Supplementary Figure S2. (a) Structure of *BORIS*. Yellow blocks represent zinc finger domain regions. The position of the siRNA target site is indicated. (**b**) siRNA-induced *BORIS* silencing efficiency was determined by western blot assay and **(c)** quantitative real-time PCR.

Supplementary Figure S3. *BORIS* silencing induced the release of cytochrome c in Caco-2 cells. The localization of cytochrome c was determined by using immunofluorescence.The right panel indicates the fold change of the signals present in immunofluorescence staining.

Supplementary Figure S4. Schematic representation of the construction of the BORIS-ZFdel plasmid. The amino acid sequence of BORIS is presented in the top panel. Zinc finger domains are highlighted in yellow. Two potential nuclear import signals reported by Jones *et al.* [1] and predicted by cNLS Mapper are shown in square frames.

Supplementary Figure S5. Immunofluorescence was applied to detect the localization of ectopically overexpressed BORIS and BORIS-ZFdel in colorectal cancer cells.

Supplementary Figure S6. 5-Aza-dc treatment up-regulated *BORIS* and decreased cell viability. Statistical differences between the control and treatments were evaluated by two-tailed Student's t-test. ***, p<0.001.

Supplementary Figure S7. Partial *BORIS* nucleotide sequence in CCD-18Co cells. The part of *BORIS* that was present did not contain mutations. The site targeted by *BORIS* siRNA is highlighted in yellow.

Supplementary Figure S8. Schedule for cell culture, transfection, 5-FU treatment, and subsequent analysis.

Supplementary Figure S9. Statistical analysis of the samples in Figure 6. The statistical differences between the samples are evaluated by two-tailed Student's t-test. *, p<0.05; **, p<0.01; ***, p<0.001.

Supplementary Figure S10. The silencing and overexpression efficiencies in Figure 6 were tested by quantitative real-time PCR.

Supplementary Table I. Primers and siRNA used in this study.

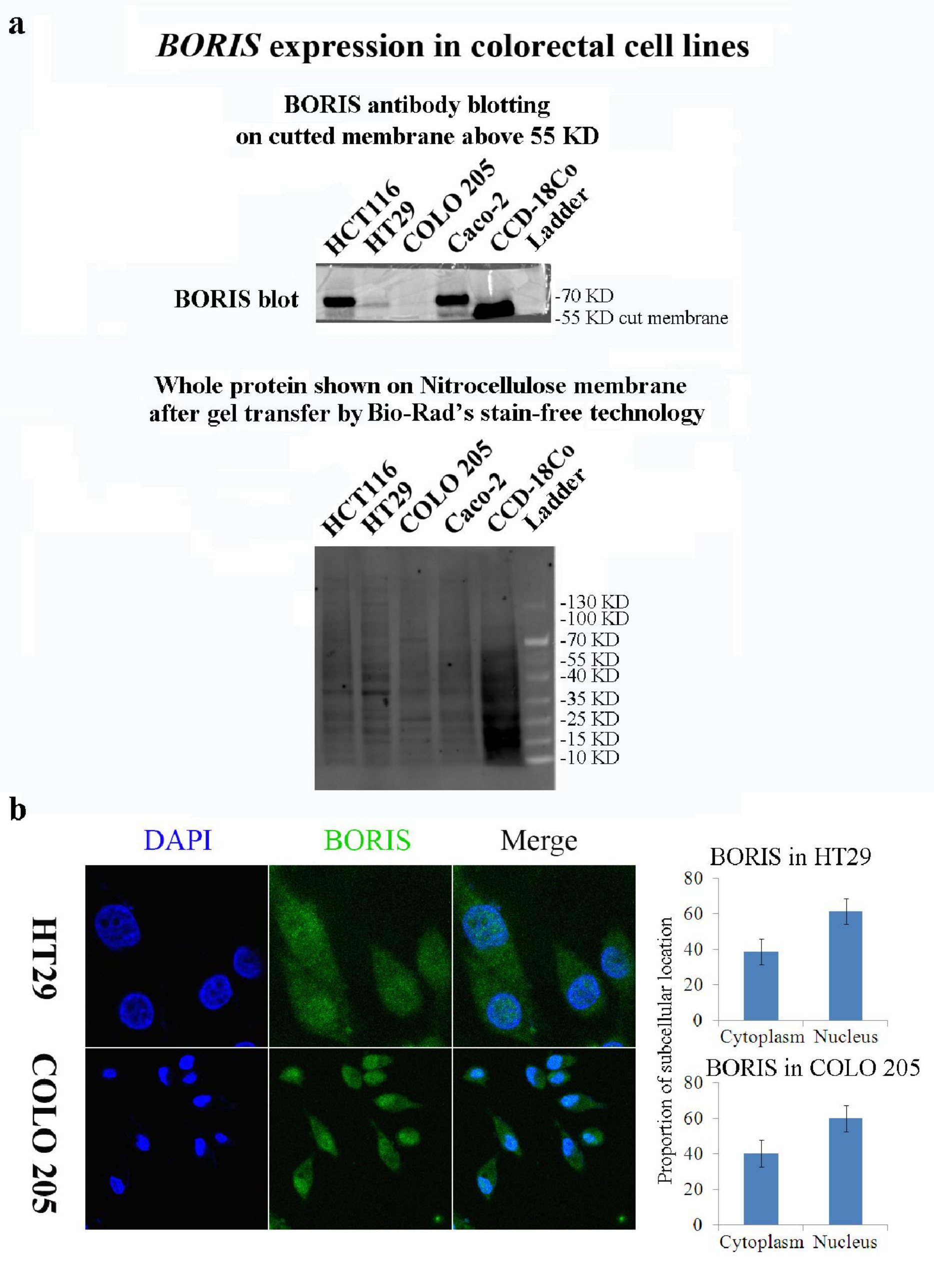

Supplementary Figure S1

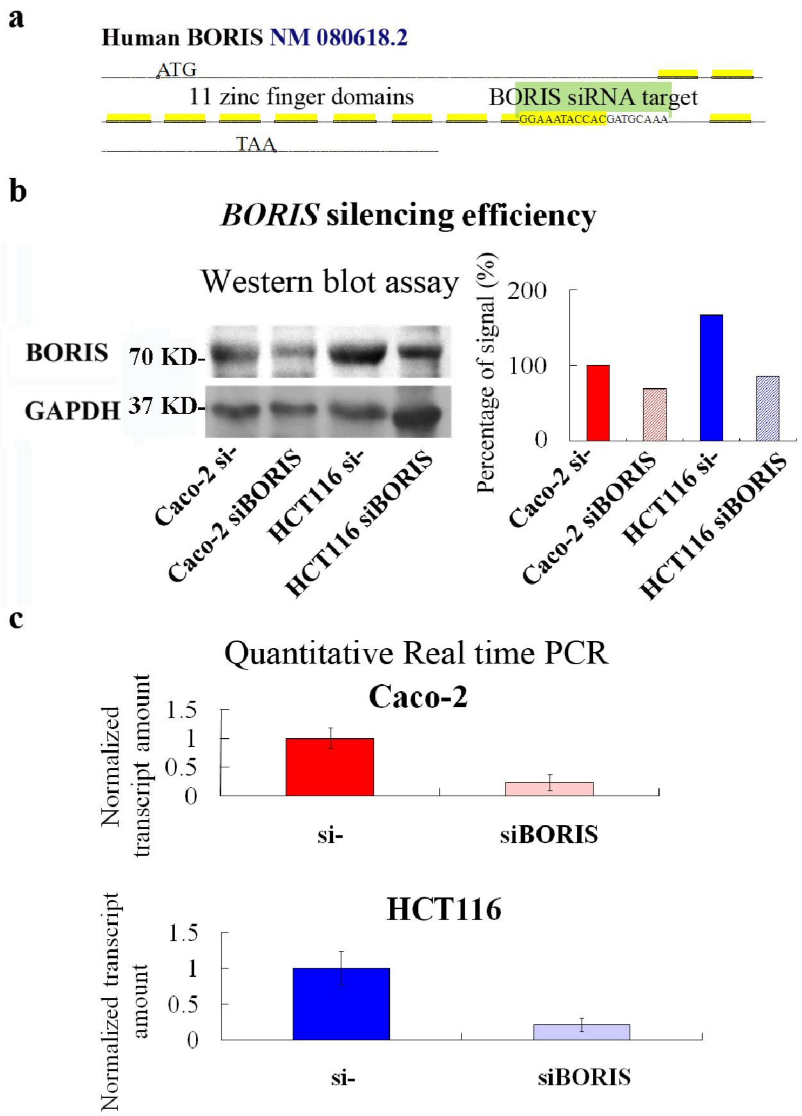

Supplementary Figure S2

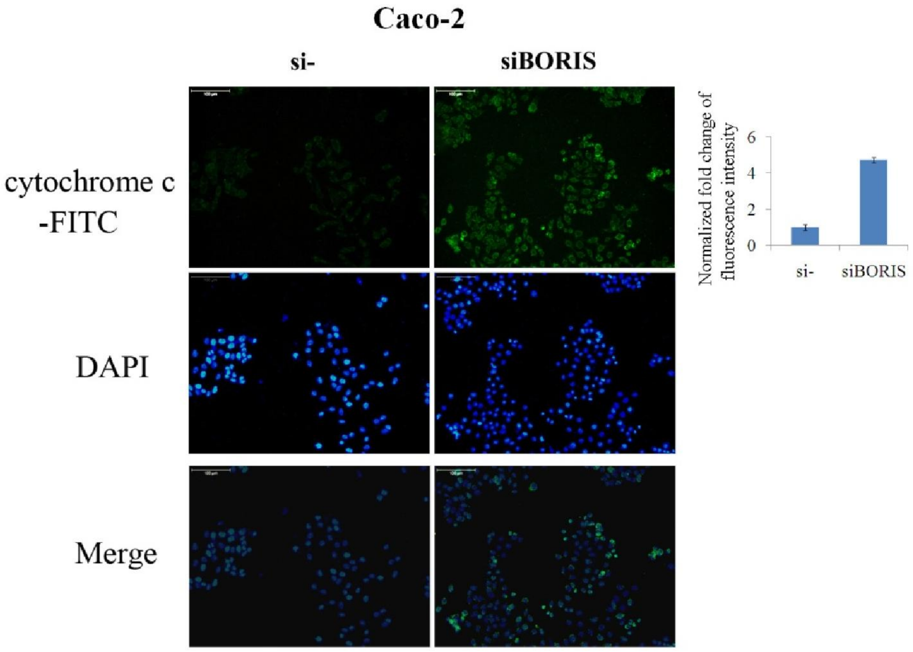

Supplementary Figure S3

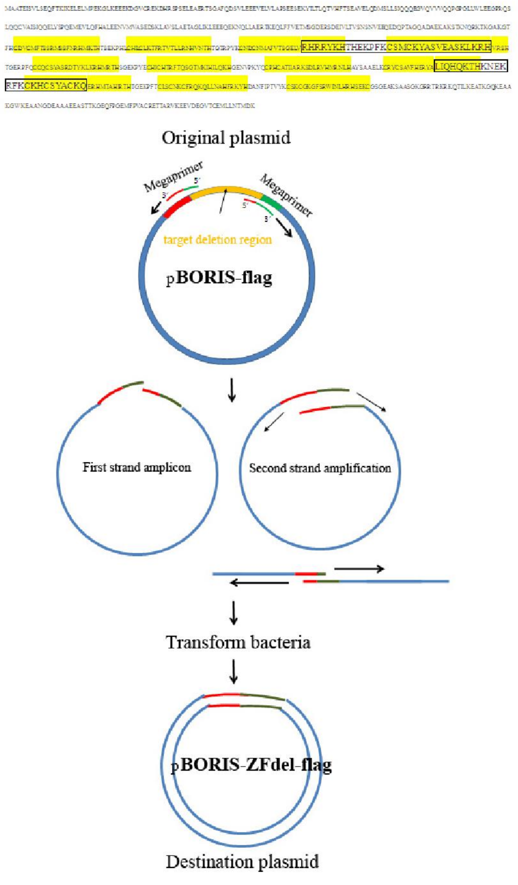

Supplementary Figure S4

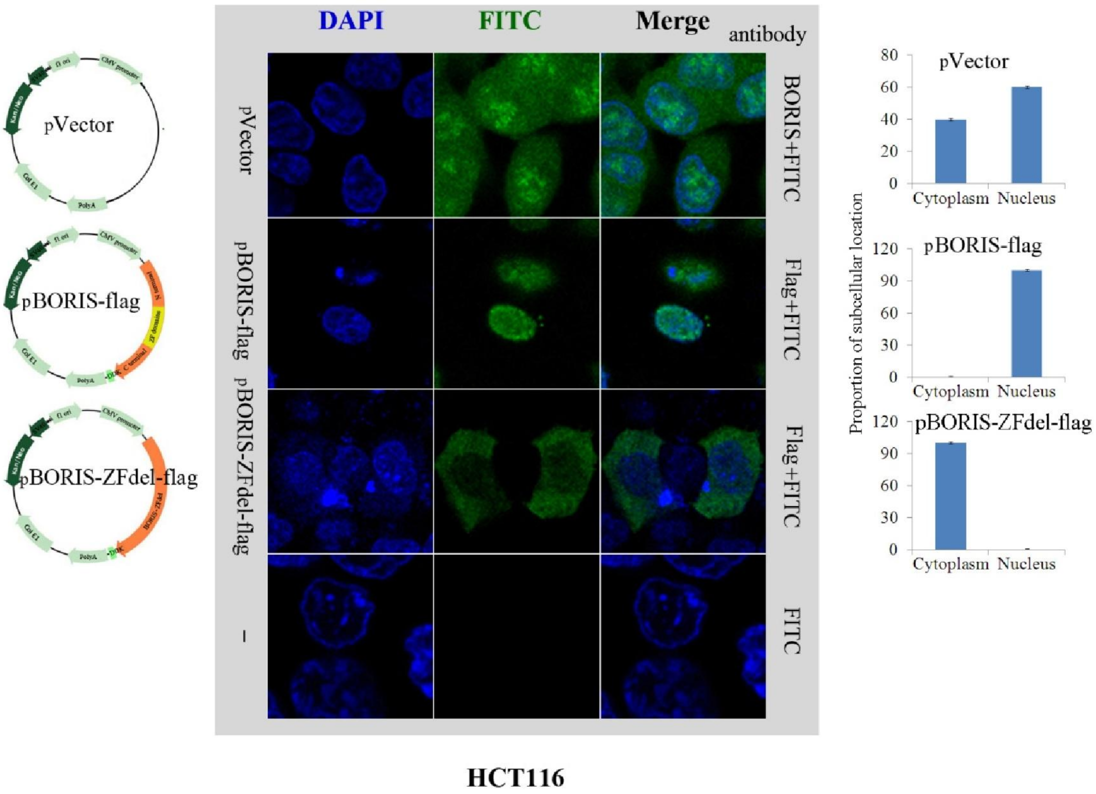

Supplementary Figure S5

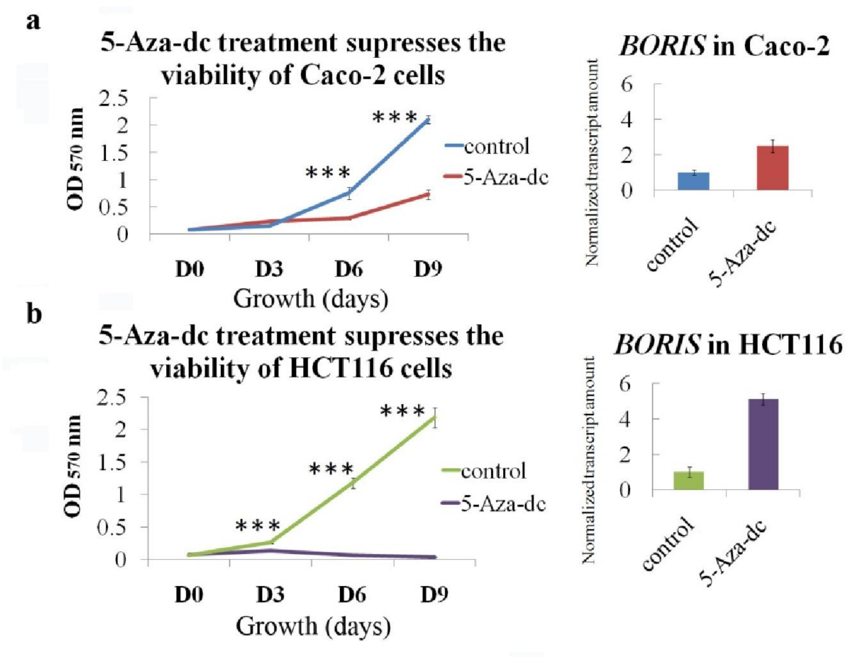

Supplementary Figure S6

CAGGCCCTACAAGTGTAACGACTGCAACATGGCATTTGTCACCAGTGGAGAACTCGTC
CGACACAGGCGCTATAAACATACTCATGAGAAACCCTTTAAATGTTCCATGTGCAAGTA
TGCCAGTGTGGAGGCAAGTAAATTGAAGCGCCATGTCCGATCCCACACTGGGGAGCGC
CCCTTTCAGTGTTGCCAGTGCAGCTATGCCAGCAGAGATACCTACAAGCTGAAACGCC
ACATGAGAACGCACTCAGGTGAGAAGCCTTACGAATGCCACATCTGCCACACCCGCTT
CACCCAGAGCGGGACCATGAAAATACATATTCTGCAGAAACACGGCGAAAATGTCCCC
AAATACCAGTGTCCCCATTGTGCCACCATCATTGCACGGAAAAGCGACCTACGTGTGC
ATATGCGCAACTTGCATGCTTACAGCGCTGCAGAGCTGAAATGCCGCTACTGTTCTGCT
GTCTTCCATGAACGCTATGCCCTCATTCAGCACCAGAAAACTCATAAGAATGAGAAGA
GGTTCAAGTGCAAACACTGCAGTTATGCCTGCAAGCAGGAACGTCATATGACCGCTCA
CATTCGTACCCACACTGGAGAGAAACCATTCACCTGCCTTTCTTGCAATAAATGTTTCC
GACAGAAGCAACTTCTAAACGCTCACTTCAGGAAATACCACGATGCAAATTTCATCCC
GACTGTTTACAAATGCTCCAAGTGTGGCAAAGGCTTTTCCCGCTGGATTAACCTGCAC
AGACATTCGGAGAAGTGTGGATCAGGGGAAGCAAAGTCGGCTGCTTCAGGAAAGGGA
AGAAGAACAAGAAAGAGGAAGCAGACCATCCTGAAGGAAGCCACAAAGGGTCAGAA
GGAAGCTGCGAAGGGATGGAAGGAAGCCGCGAACGGAGACGAAGCTGCTGCTGAGG
AGGCTTCCACCACGAAGGGAGAACAGTTCCCAGGAGAGATGTTTCCTGTCGCCTGCA
GAGAAACCACAGCCAGAGTCAAAGAGGAAGTGGATGAAGGCGTGACCTGTGAAATG
CTCCTCAACACGATGGATAA

Supplementary Figure S7

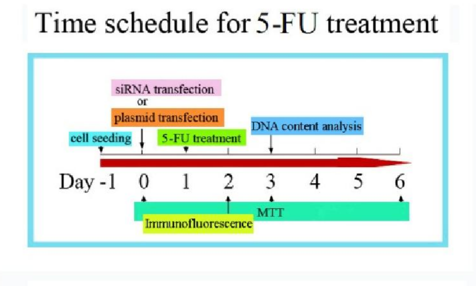

Supplementary Figure S8

Statistical analyses of Figure 6a

	si- plus DMSO		si- plus 0.6 uM 5-FU		si- plus 1.2 uM 5-FU		siBORIS plus DMSO		siBORIS plus 0.6 uM 5-FU	
	D3	D6	D3	D6	D3	D6	D3	D6	D3	D6
si- plus 0.6 uM 5-FU	**	--								
si- plus 1.2 uM 5-FU	***	*	*	***						
siBORIS plus DMSO	***	*	***	***	***	**				
siBORIS plus 0.6 uM 5-FU	***	*	***	***	***	***	--	***		
siBORIS plus 1.2 uM 5-FU	***	*	***	***	***	***	**	***	*	--

Statistical analyses of Figure 6b

	pVector plus DMSO		pVector plus 0.6 uM 5-FU		pVector plus 1.2 uM 5-FU		pBORIS plus DMSO		pBORIS plus 0.6 uM 5-FU	
	D3	D6	D3	D6	D3	D6	D3	D6	D3	D6
pVector plus 0.6 uM 5-FU	**	***								
pVector plus 1.2 uM 5-FU	**	***	--	***						
pBORIS plus DMSO	***	***	***	***	***	***				
pBORIS plus 0.6 uM 5-FU	***	--	***	***	***	***	*	**		
pBORIS plus 1.2 uM 5-FU	**	***	***	***	***	***	*	***	--	**

Statistical analyses of Figure 6c

	DMSO plus pVector		DMSO plus pBORIS-ZFdel		5-FU 0.6 uM plus pVector		5-FU 0.6 uM plus pBORIS-ZFdel		H_2O_2 500 uM plus pVector	
	D4	D7	D4	D7	D4	D7	D4	D7	D4	D7
DMSO plus pBORIS-ZFdel	--	--								
5-FU 0.6 uM plus pVector	***	***	***	***						
5-FU 0.6 uM plus pBORIS-ZFdel	***	***	***	***	–	--				
H_2O_2 500 uM plus pVector	***	***	***	***	**	**	--	**		
H_2O_2 500 uM plus pBORIS-ZFdel	***	***	***	***	***	***	*	***	--	--

Supplementary Figure S9

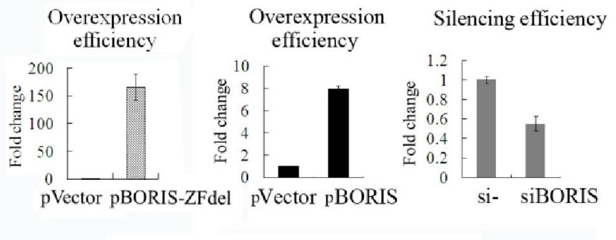

Supplementary Figure S10

Supplementary Table I. Primers and siRNA used in this study.

Primer/siRNA	Forward (5'to3')	Reverse (5'to3')	application
BORIS	AGTGAGAAGCCTCACCTGTGTC	GACGAGTTCTCCACTGGTGACA	Realtime PCR
GAPDH	CCCACTCCTCCACCTTTGAC	TGTTGCTGTAGCCAAATTCGT	Realtime PCR
actin	AAAATCTGGCACCACACCTTC	TAGCACAGCCTGGATAGCAA	Realtime PCR
BRCA1	ACTCTGAGGACAAAGCAGCG	CATCCCTGGTTCCTTGAGGG	Realtime PCR[2]
c-Myc	GCTGCTTAGACGCTGGATTT	CACCGAGTCGTAGTCGAGGT	Realtime PCR[3]
BORIS-sequence-primer	CAGGCCCTACAAGTGTAACGACTGCAA	CCCAAGCTTTGGCTTATCCATCGTGTT	Sequence
Negative siRNA	UUCUCCGAACGUGUCACGUdTdT	ACGUGACACGUUCGGAGAAdTdT	Knockdown
BORIS siRNA	GGAAAUACCACGAUGCAAATT	UUUGCAUCGUGGUAUUUCCtt	Knockdown[4]
Megaprimer	AAGGAACCGGATCAGGGGAAGCAAAGT CGGCTGCTT	CCTGATCCGGTTCCTTTTGCTCCCTTTGT CTTTC	construction of BORIS-ZFdel

References

1. Jones, T. A. et al. Widespread expression of BORIS/CTCFL in normal and cancer cells. *PLoS One* **6**, e22399, doi:10.1371/journal.pone.0022399 (2011).
2. He, X. & Zhang, P. Serine/arginine-rich splicing factor 3 (SRSF3) regulates homologous recombination-mediated DNA repair. *Molecular cancer* **14**, 158, doi:10.1186/s12943-015-0422-1 (2015).
3. Zou, L. et al. Down-regulated PLAC8 promotes hepatocellular carcinoma cell proliferation by enhancing PI3K/Akt/GSK3beta/Wnt/beta-catenin signaling. *Biomedicine & pharmacotherapy = Biomedecine & pharmacotherapie* **84**, 139-146, doi:10.1016/j.biopha.2016.09.015 (2016).
4. Dougherty, C. J. et al. Selective apoptosis of breast cancer cells by siRNA targeting of BORIS. *Biochemical and biophysical research communications* **370**, 109-112, doi:10.1016/j.bbrc.2008.03.040 (2008).
